# Warriors, Worriers, and COVID-19: An Exploratory Study of the Catechol O-Methyltransferase Val158Met Polymorphism Across Populations

**DOI:** 10.7759/cureus.10103

**Published:** 2020-08-28

**Authors:** Ravi P Rajkumar

**Affiliations:** 1 Psychiatry, Jawaharlal Institute of Postgraduate Medical Education and Research, Pondicherry, IND

**Keywords:** covid-19, health anxiety, genetics, catechol o-methyltransferase gene

## Abstract

Background

Prevalence and mortality rates during the coronavirus disease 2019 (COVID-19) pandemic have varied widely across nations. This phenomenon may be partly due to regional variations in health-related behaviours, some of which may be influenced by health anxiety. A functional polymorphism of the catechol O-methyltransferase (COMT) gene, designated rs4680 or Val^158^Met, has been associated with anxiety-related behaviours and the so-called "worrier" phenotype.

Methods

In this exploratory study, an analysis of the correlation between the frequencies of the Met allele of the COMT gene across 28 countries, obtained from the public domain Allele Frequency Database (ALFRED), and the COVID-19 prevalence and mortality rates in these countries, obtained from the Johns Hopkins Medical University web-based dashboard, was carried out while controlling for population size and median age in each country.

Results

Allele frequencies varied widely across populations. Met allele frequency was positively correlated with COVID-19 prevalence (ρ = 0.527, p = 0.004) and mortality rate (ρ = 0.542, p = 0.003) across nations. However, this correlation was no longer significant after controlling for confounders.

Conclusions

These preliminary results suggest that there may be a relationship between the COMT Val^158^Met or rs4680 functional polymorphism and the impact of COVID-19 across nations, which could plausibly be mediated by maladaptive anxiety-related behaviours.

## Introduction

Despite the global scope of the acute respiratory illness (COVID-19) caused by the severe acute respiratory syndrome coronavirus 2 (SARS-CoV-2) strain, prevalence and mortality rates for this condition have varied widely across nations. While patterns of international travel may partially explain the former, the same is not true of the latter [1]. A variety of explanations for this phenomenon have been given, including variation in the median age of the population, the presence of particular medical comorbidities, past exposure to other coronavirus strains, implementation of containment measures, and levels of sociability and institutional trust. However, none of these has been convincingly demonstrated [1-3]. Nevertheless, given our knowledge of the routes of transmission of COVID-19, the available evidence makes it clear that behavioural and social factors play a central role in either accelerating or curtailing the spread of this disease [4]. Moreover, the direct and indirect social and economic effects of the COVID-19 outbreak have led to psychological distress and symptoms of anxiety or depression. Reduced adherence to hygiene or other preventive measures has been associated with depressive symptomatology [5,6].

Health anxiety is a critical concept in understanding psychological responses to infectious disease outbreaks [7]. In this context, "health anxiety" refers not only to the psychiatric disorder also known as hypochondriasis, but to a multifaceted trait which encompasses fear of or preoccupation with illness, as well as a tendency to interpret bodily sensations or changes as symptoms of illness [7-9]. From an evolutionary point of view, health anxiety can be understood as part of the "behavioural immune system," a set of behaviours that have evolved to reduce the risk of exposure to infectious pathogens [10]. However, excessive health anxiety can lead to maladaptive behaviours which may paradoxically increase the spread of infectious diseases; these include avoiding health care when symptomatic, frequent clinic visits at times of community transmission, hoarding protective material resulting in a shortage or resorting to unproven methods of prevention or treatment [7].

Little is known about the genetic and biological mechanisms that underlie this complex cognitive and behavioural response. However, the rs4680 functional polymorphism of the catechol-O-methyltransferase (COMT) gene, commonly designated COMT Val158Met, merits closer examination in this context. This polymorphism is characterized by a single base substitution (G→A) at codon 158 of the COMT gene, leading to the substitution of methionine (Met) for valine (Val) and causing decreased enzymatic degradation of dopamine, particularly in the prefrontal cortex. The result of this change appears to be heightened cognitive performance at the cost of greater levels of anxiety and vulnerability to stress, while the wild-type (Val) gene is associated with lower cognitive performance but better emotional regulation and stress resilience [11,12]. The existence of this variation has led to the proposal of the "warrior/worrier hypothesis", which associates specific strategies linked to a survival advantage with each genetic variant: the "warrior" (Val) allele conferring an advantage in the rapid processing of aversive stimuli, and the "worrier" (Met) allele conferring an advantage on tasks involving memory and sustained attention [12,13]. In line with this, the Met ("worrier") allele has been significantly associated with anxiety disorders in some samples. However, this relationship appears to be moderated by factors such as age, gender, ethnicity and adverse life events [14,15]. Contrasting findings have been reported in some studies, with the Val ("warrior") allele being associated with anxiety-related constructs such as neuroticism and harm avoidance [16].

In the context of the COVID-19 pandemic and its attendant psychosocial stressors, it is plausible to hypothesize that the "worrier" allele might be associated with excessive health anxiety and maladaptive avoidance or safety behaviours, which could, in turn, have a detrimental effect on disease spread. The current study aimed to test this hypothesis at a primary level by examining the relationship between existing data on the frequency of the Met allele across different populations, and indices of the impact of COVID-19 on the prevalence, mortality and case-fatality rates in these populations.

## Materials and methods

Data on the frequencies of the Val and Met alleles of the COMT rs4680 polymorphism were obtained from the Allele Frequency Database (ALFRED), a public domain repository of genetic information for academic and research purposes [17]. This information was available for a total of 30 populations to which a single nationality could be assigned. Information on the relative frequencies of each genotype (Met-Met, Val-Met or Val-Val) across populations was not available.

Information on the total number of cases, recovered cases, and deaths for each nation was obtained on June 23, 2020, from the Johns Hopkins Medical University’s web-based dashboard, which provides real-time information on these parameters [18]. This data was used to calculate: (a) the prevalence rate, defined as the total number of cases per 1 million population, (b) the crude mortality rate, defined as the total number of deaths attributed to COVID-19 per 1 million population, and (c) the case fatality rate, defined as the ratio of deaths to total cases with an outcome. Of the 30 nations for which allele frequency data were available, two countries, Cambodia and Vietnam, had no reported deaths due to COVID-19, precluding the computation of mortality or fatality rates. Hence, these two countries were excluded from the analysis, leaving 28 countries for which complete data was available: Belarus, China, Colombia, Denmark, Estonia, Finland, France, Ghana, Hungary, Ireland, Italy, Japan, Kazakhstan, Kuwait, Kyrgyzstan, Malaysia, Mexico, Moldova, Peru, The Russian Federation, Slovenia, the Republic of Korea, Spain, Tunisia, Turkey, The United Kingdom, Ukraine and Uzbekistan.

Data analysis was carried out using IBM's Statistical Package for Social Sciences, version 20 (SPSS version 20, IBM Corporation). Study variables were tested for normality using the Shapiro-Wilk test. COMT allele frequencies were normally distributed, but none of the COVID-19 indices were; therefore, Spearman’s rank correlation coefficient (ρ) was used to test for the presence of a monotonic association between Met allele frequency and the prevalence, mortality rate, and case fatality rate for COVID-19 in each nation. All tests were two-tailed. Owing to the small study sample size and the exploratory nature of this study, a conservative significance value of p < 0.01 was selected for all bivariate analyses.

To correct for the potential confounding effects of population size and median age for each nation, both of which can influence the computed outcome measures, partial correlation analyses were carried out for all associations that emerged as significant in the bivariate analyses, controlling for the effects of these variables [1,2,19]. Data on populations for each nation was obtained from World Bank statistics, and data on median age was obtained from the Central Intelligence Agency’s World Fact Book [20,21].

## Results

There was significant variation in the frequency of the Met allele across different populations, ranging from a minimum frequency of 0.252 in a South Korean sample to a maximum of 0.609 in a Danish sample.

Bivariate correlation analyses found a significant positive correlation between Met allele frequency for each nation-level sample and both the prevalence (ρ = 0.527, p = 0.004) and mortality rate (ρ = 0.542, p = 0.003) due to COVID-19 in their respective nations. No significant association was found between Met allele frequencies and the case fatality rate per nation (ρ = 0.133, p = 0.517).

The various indices of the impact of COVID-19 in each nation were significantly correlated with each other as follows: prevalence and mortality (ρ = 0.811, p < 0.001), mortality and case fatality (ρ = 0.542, p = 0.004). There was no significant relationship between prevalence and case fatality rates.

Analysis of confounding factors confirmed the earlier reports of a significant relationship between median age and the case fatality rate, though only at a trend level (ρ = 0.424, p = 0.032), but found no relationship between population size and either prevalence (ρ = -0.149, p = 0.446), mortality (ρ = -0.020, p = 0.572) or case fatality rates (ρ = 0.164, p = 0.42). Met allele frequency was itself negatively correlated with population size at a trend level (ρ = -0.402, p = 0.034) but showed no significant correlation with median age (ρ = 0.292, p = 0.131).

On partial correlation analyses, the association between the COMT rs4680 Met allele and the prevalence of COVID-19 remained significant at a trend level after correcting for median age and population size (partial r = 0.348, p = 0.096). However, the association with the mortality rate was no longer significant (partial r = 0.170, p = 0.428).

## Discussion

The above results suggest that a positive correlation may exist between population frequencies of the COMT rs4680 Met (“worrier”) allele and the spread of COVID-19 in different populations. This observation must be tempered by the fact that this association was only marginally significant when correcting for the known confounding factors of population size and median age. No significant relationship was found between “worrier” allele frequency and the case fatality rate, which may reflect the fact that this variable appears to be more influenced by other factors, such as age, medical comorbidities, and variations in individual immune-inflammatory responses [19].

If the relationship identified above truly exists, it may be mediated through excessive levels of health anxiety and resultant maladaptive behaviours, which could have the unintended effect of accelerating or failing to curtail the spread of COVID-19. A related possibility, based on the observation that the “worrier” allele is associated with poorer stress tolerance [11], could be that such maladaptive anxiety occurs in response to the stress induced by the COVID-19 outbreak and the measures necessary for its containment [22]. Alternately, the “warrior” allele may confer an advantage in terms of threat perception, leading to better adherence to protective behaviours and minimizing the spread of the disease.

Though such a hypothesis is speculative, it gains credibility from research suggesting that other genetic variants related to anxiety vulnerability, such as the serotonin-transporter-linked polymorphic region (5-HTTLPR), may have been subjected to selection pressures caused by infectious pathogens [23]. In other words, there may be a wide array of genetic polymorphisms involving neurotransmitter function, and which confer a survival advantage in terms of shaping adaptive responses to the threat of outbreaks of infectious disease [10,24].

These results must be viewed in light of several limitations. First, they are based on allele frequency data from relatively small samples, which may not accurately estimate the true frequencies at a national level. Second, the indices of the severity of COVID-19 used in this study are influenced by a multitude of variables, any of which could conceivably act as a confounding factor. Third, owing to the cross-sectional nature of this analysis, this study fails to account for the possibility that the estimated prevalence and mortality rates may change substantially in certain countries as the pandemic continues to spread. Fourth, the association between COMT functional polymorphisms and anxiety, though grounded in theoretical and experimental work, has never been conclusively demonstrated in the context of health anxiety or responses to the threat of infection. Fifth, allele frequency data were available only for a relatively small number of countries. Finally, in exploratory research of this kind, the likelihood of false-positive results always exists, though measures were taken to minimize this risk.

## Conclusions

In conclusion, the results of the above analysis suggest that there may be an association between the COMT rs4680 “worrier” polymorphism and certain measures of the impact of COVID-19 across nations. Such associations need to be verified prospectively and in larger samples, as they may shed light on the genetic and biological mechanisms which influence behavioral responses to a pandemic.
